# rAAV9 vector biodistribution in nonhuman primate brain and spinal cord following lumbar intrathecal infusion

**DOI:** 10.3389/fmed.2026.1819594

**Published:** 2026-04-29

**Authors:** Emdadul Haque, Nino Devidze, Suku Nagendran, Rumana Haque-Ahmed, Sean McAuliffe, Alain Lamontagne, John Ashkenas, Steven J. Gray, Fred Porter

**Affiliations:** 1 Taysha Gene Therapies, Dallas, TX, United States; 2 EquiPoise Communication, Toronto, ON, Canada; 3 Department of Pediatrics, University of Texas Southeastern Medical Center, Dallas, TX, United States

**Keywords:** AAV9 vectors, biodistribution, brain, gene therapy, intracisternal injection, intrathecal injection, nonhuman primate

## Abstract

**Introduction:**

Delivery of recombinant adeno-associated virus serotype 9 (rAAV9) into cerebrospinal fluid bypasses the blood–brain barrier and can permit efficient brain tissue transduction. Whereas intracisterna magna injection (ICM) and other routes have been considered, lumbar intrathecal (IT) delivery offers a minimally invasive approach for treating genetic disorders of the central nervous system. To evaluate central nervous system biodistribution, we quantified vector genome (vg) copies of five rAAV9 vectors in cynomolgus macaques following ICM or lumbar IT delivery.

**Methods:**

A total of 48 animals were followed across five studies: four lumbar IT infusion studies with four recombinant adeno-associated virus serotype 9 vectors (TSHA-101, -102, -105, and -120); and one ICM infusion study (TSHA-102 only). Stock vectors were diluted to dosing concentrations and 2.5 mL were administered to each animal. After animals were sacrificed, tissue samples were harvested and biodistribution was assessed via qPCR.

**Results:**

Despite protocol differences specifying different doses (human effective dose range, 2.6 × 10^13^ to 2.0 × 10^15^ vg), necropsy times (1 month to 1 year), and central nervous system tissues harvested, rAAV9 DNA levels in the brain and spinal cord were generally dose-dependent and consistent across tissues. At comparable doses and times, IT and ICM delivery led to widespread and consistent distribution of vector genomes, approximating 1 vg/diploid host genome throughout the brain at the higher doses tested.

**Conclusion:**

These findings support IT administration as an effective, minimally invasive approach to central nervous system-directed gene therapy.

## Graphical abstract

**Figure:**
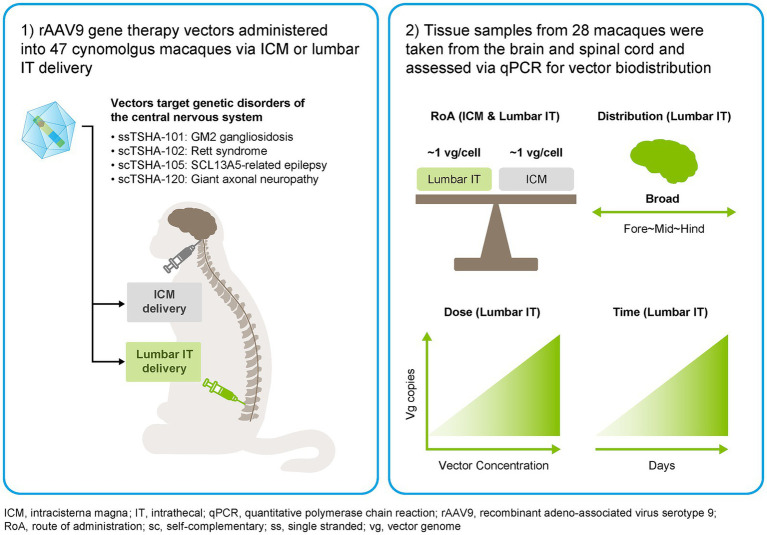

## 1 Introduction

Intrathecal (IT) administration of recombinant adeno-associated viral (rAAV) vectors by lumbar puncture is one of several routes of administration (RoAs) that have been explored for delivering therapeutic transgenes directly to the central nervous system (CNS) (1). As with intracisterna magna (ICM) or intra-cerebroventricular (ICV) instillation, IT delivery bypasses the blood–brain barrier, reduces vector load in systemic circulation, and minimizes risk of interference of pre-existing anti-AAV antibodies (2, 3). Compared to other CNS-directed approaches (Supplementary Figure S1), the IT route may be preferred as a minimally invasive approach for clinical use (2).

Initial studies from Gray et al. (4) in cynomolgus macaques (nonhuman primates; NHPs) established that IT delivery of rAAV9 reaches all regions of the brain and spinal cord. With the low-input doses that were typical at the time of that study [e.g., 1.8 × 10^12^ vector genomes (vg)/animal; human equivalent dose (HED): 1.6 × 10^13^ vg], brain transduction levels were modest, corresponding to approximately ≥0.01 (vg) detected, on average, per host diploid genome (DG) in the brain parenchyma. Nevertheless, DNA analysis showed quantifiable vector genome levels across brain and spinal cord tissues.

Subsequently, other doses and RoAs have been explored in large animal models (2, 5), and multiple gene therapy (GT) vectors have moved into the clinic, including several that require CNS access to correct brain cell abnormalities. To date, lumbar IT delivery remains the most common RoA for this purpose (6). Since 2020, we have investigated various rAAV9 candidate GT vectors for rare and ultrarare CNS-related genetic disorders, of which four have advanced to toxicology/biodistribution studies in NHPs, including studies of TSHA-120 (scAAV9/JeT-GAN) conducted prior to 2020 by Dr. Gray’s laboratory. The four vectors assessed in this study (Table 1) carry different therapeutic transgenes, for the treatment of Tay–Sachs disease (β-hexosaminidase A deficiency), Rett syndrome (RTT) (associated with loss-of-function mutations in *MECP2*), *SLC13A5*-related epilepsy, and giant axonal neuropathy (GAN)—all conditions for which GT must be targeted broadly to the brain parenchyma. Here, we summarize biodistribution of these four vector constructs within the NHP CNS, based on data from five studies. Four of the studies utilized lumbar IT administration, while the TSHA-102 vector was also evaluated following ICM administration.

**Table 1:** Gene therapy vectors studied in NHPs.

Construct	Vector structure^a^	Neurodevelopmental /neurodegenerative disorder	Therapeutic transgene	Route of administration
TSHA-101 (24) ssAAV9-CAG-*HEXBP2AHEXA*	ssAAV9	GM2 gangliosidosis	HEXA	Lumbar IT
TSHA-102 (7, 22) scAAV9-MeP426-*miniMECP2*-miRARE	scAAV9	Rett syndrome	MeCP2	Lumbar IT
				ICM
TSHA-105 scAAV9-UsP-*hSLC13A5*opt-SpA	scAAV9	SLC13A5-related epilepsy	SLC13A5	Lumbar IT
TSHA-120^b^ (25, 26) scAAV9/JeT-GAN	scAAV9	Giant axonal neuropathy (GAN)	Gigaxonin	Lumbar IT

a Single-stranded (ss) or self-complementary (sc).

b TSHA-120 is also called scAAV9/JeT-GAN (26).

Because the vectors differed with respect to genomic structure (i.e., self-complementary versus single-stranded AAV9) and transgene regulation (including selection of promoters to drive brain expression and inclusion of negative regulatory sequences to prevent overexpression), cross-study comparison of gene or protein expression was not expected to be fruitful. Intrinsic differences in stability of transgene mRNAs or encoded proteins could further complicate analysis of downstream events. Therefore, the current comparative analysis was restricted to vector biodistribution.

## 2 Materials and methods

Animals were conditioned and acclimated prior to study start date. All NHPs were wild-type, young (~1.5–4.5-year-old) *Macaca fascicularis* weighing ~1.5–3.7 kg on Day 0 of each study. Serum AAV9 neutralizing (nAb) antibodies, were assessed before treatment. Animals with detectable nAbs (e.g., titers ≥1:10) were preferentially chosen for diluent-only treatment, whereas those with low or undetectable nAbs could receive rAAV9 treatment.

Virions were introduced into the CNS by IT or ICM injection, under anesthesia. TSHA-101, TSHA-102, and TSHA-105 studies were directed by Taysha’s current and former employees and carried out by Lovelace Biomedical (Albuquerque, NM). Injections (2.5 mL total volume) were performed with animals prone or recumbent, not in the Trendelenburg position. IT injection was to the L4-5 or L5-6 intervertebral space, with the needle tip angled slightly cranially. ICM injection to the suboccipital space was performed with animals prone, their heads tipped forward; the needle was introduced at a perpendicular angle. For both target sites, needle placement was confirmed by return of CSF. No immunosuppression was used. The TSHA-120 studies were planned and coordinated by Dr. Steven Gray’s laboratory at the University of North Carolina at Chapel Hill and carried out by MPI Research (Mattawan, MI), along with Dr. Gray’s laboratory. Each study was approved by a local Institutional Animal Care and Use Committee.

TSHA-101 was manufactured by Andelyn Biosciences (Columbus, OH). Titer concentration was measured by quantitative polymerase chain reaction (PCR). TSHA-102 and TSHA-105 were manufactured by Catalent Cell and Gene Therapy (BWI, Maryland). The titer was measured by droplet digital PCR. The manufacturing procedure has been previously reported (7). TSHA-120 was manufactured by the University of North Carolina Vector Core, by triple transfection of HEK293 cells according to published methods (8) and titered by dot blot. Since the studies on the various rAAV9 vectors occurred independently, at different times and different laboratories, vector titration and vector biodistribution were not normalized across studies.

rAAV9 stock solutions for studies involving TSHA-101, TSHA-102, and TSHA-105 were diluted in phosphate-buffered saline (PBS) with 5% sorbitol, 0.001% Poloxamer 188 [pH 7.4 ± 0.1 (Catalent Cell and Gene Therapy)] and administered sterilely at a consistent volume of 2.5 mL. rAAV9 for the TSHA-120 vector was prepared in PBS containing 5% sorbitol (University of North Carolina Vector Core) and injected in a 1 mL injection volume. For TSHA-101, the diluent was 20 mM Tris (pH 8.0), supplemented with 200 mM NaCl, 1 mM MgCl2, and 0.001% (w/v) poloxamer 188. NHP tissues from the TSHA-101, TSHA-102, and TSHA-105 studies were homogenized and lysed, and lysates were loaded to QIASymphony (Qiagen) for automated DNA extraction. NHP tissue from the TSHA-120 study was purified using a Qiagen DNeasy blood and tissue kit using a Qiagen QiaCube. In all cases, vector stocks prepared in the appropriate diluent were evaluated to ensure that they were within prespecified limits of titer loss, impurities, stability and potency.

qPCR was used to quantify rAAV9 DNA in spinal cord or brain homogenates prepared from prespecified tissue samples. Vector genome copy number is reported per microgram of *M. fascicularis* DNA and per *M. fascicularis* DG. The latter approach, which normalizes rAAV9 copies to cell number in the CNS samples, is best suited to cross-species comparisons of vector biodistribution.

In addition to differences in RoA and the preparative procedures noted above, salient protocol differences across NHP experiments are identified in Table 2. These included: time to necropsy (ranging from 30 days in the TSHA-101 study to 1 year in the TSHA-120 study, with two time points, 90 and 180 days, used in some studies); selection of brain tissues for rAAV9 DNA quantification (serial sections across brain slabs in a total of 15 brain sections from a series of five coronal slabs along a rostro-caudal axis in the TSHA-120 study versus finer dissection of two to six brain sites in other studies); and harvest of brain tissues only versus brain and spinal cord tissues. These cross-study protocol differences may confound the attempt to quantify biodistribution with the various rAAV9 constructs used.

**Table 2:** Dosing and analysis of rAAV9 vectors across five NHP biodistribution studies.

Construct	RoA	Dose per animal (vg)	Human equivalent dose (vg)^a^	Time to necropsy (days post-treatment)			
				30	90	180	364
TSHA-101^b^	IT	5.8 × 10^13^	5.0 × 10^14^	**2M, 2F**			
TSHA-102^c^	IT	2.9 × 10^13^	2.5 × 10^14^		3F	3F	
		5.8 × 10^13^	5.0 × 10^14^		**3F**	**3F**	
		2.3 × 10^14^	2.0 × 10^15^		3F	3F	
TSHA-102	ICM	2.3 × 10^14^	2.0 × 10^15^		**3F**		
TSHA-105^c^	IT	5.8 × 10^13^	5.0 × 10^14^		2M, 2F	2M, 1F	
		2.3 × 10^14^	2.0 × 10^15^		**2M, 2F**	**2M, 1F**	
TSHA-120	IT	3.0 × 10^12^	2.6 × 10^13^				**2M, 2F**
		1.2 × 10^13^	1.0 × 10^14^				**2M, 2F**

Tissues from animals indicated in bold were examined by qPCR to quantify CNS distribution of the rAAV9 vector. Vehicle-treated animals in each study (*N* = 20 in total) are not shown.

CSF, cerebrospinal fluid; CNS, central nervous system; ICM, intracisterna magna administration; F, female; IT, intrathecal administration; M, male; NHP, nonhuman primate; qPCR, quantitative polymerase chain reaction; rAAV9, recombinant adeno-associated virus serotype 9; RoA, route of administration; vg, vector genome.

a Estimated based on the ratio of CSF volumes of NHPs versus humans, as reported by Emami et al. (9).

b Four additional animals, treated with TSHA-101 along with immunosuppressants, are not included in this analysis.

c Per study protocol, of three doses of TSHA-102 tested in this study (2.9 × 10^13^, 5.8 × 10^13^, and 2.3 × 10^14^ vg/animal, *n* = 6 for each dose), only the intermediate dose was examined to quantify vector biodistribution. Similarly, one of two doses of TSHA-105 (*N* = 7 for each dose) was examined for biodistribution.

### 2.1 Statistical analysis

Given that all comparisons described here are based on small numbers of animals, descriptive statistics are used throughout, and no sub-analysis was attempted (e.g., by sex of the treated animals). Vector biodistribution values for specific CNS tissues are reported as mean vg/μg DNA across equivalently treated animals, with standard deviations as shown. Estimates of HED were calculated on the basis of relative CSF volume, which is estimated to be ~8.7 times greater in humans than in cynomolgus macaques (9).

## 3 Results

Five NHP studies included 71 cynomolgus macaques, of which 47 animals were treated with a single dose of TSHA-101, -102, -105, or -120 at varying doses (Table 1). Of these rAAV9-treated animals, 28 were analyzed for vector biodistribution (Table 2), along with 20 control animals treated with vehicle alone. None of the studies had control animals with quantifiable levels of rAAV9 vector genome, with the exception of the TSHA-120 study, in which some negative control samples showed low-level contamination.

### 3.1 TSHA-101 CNS biodistribution at 30 days post-lumbar IT administration

**Figure 1:**
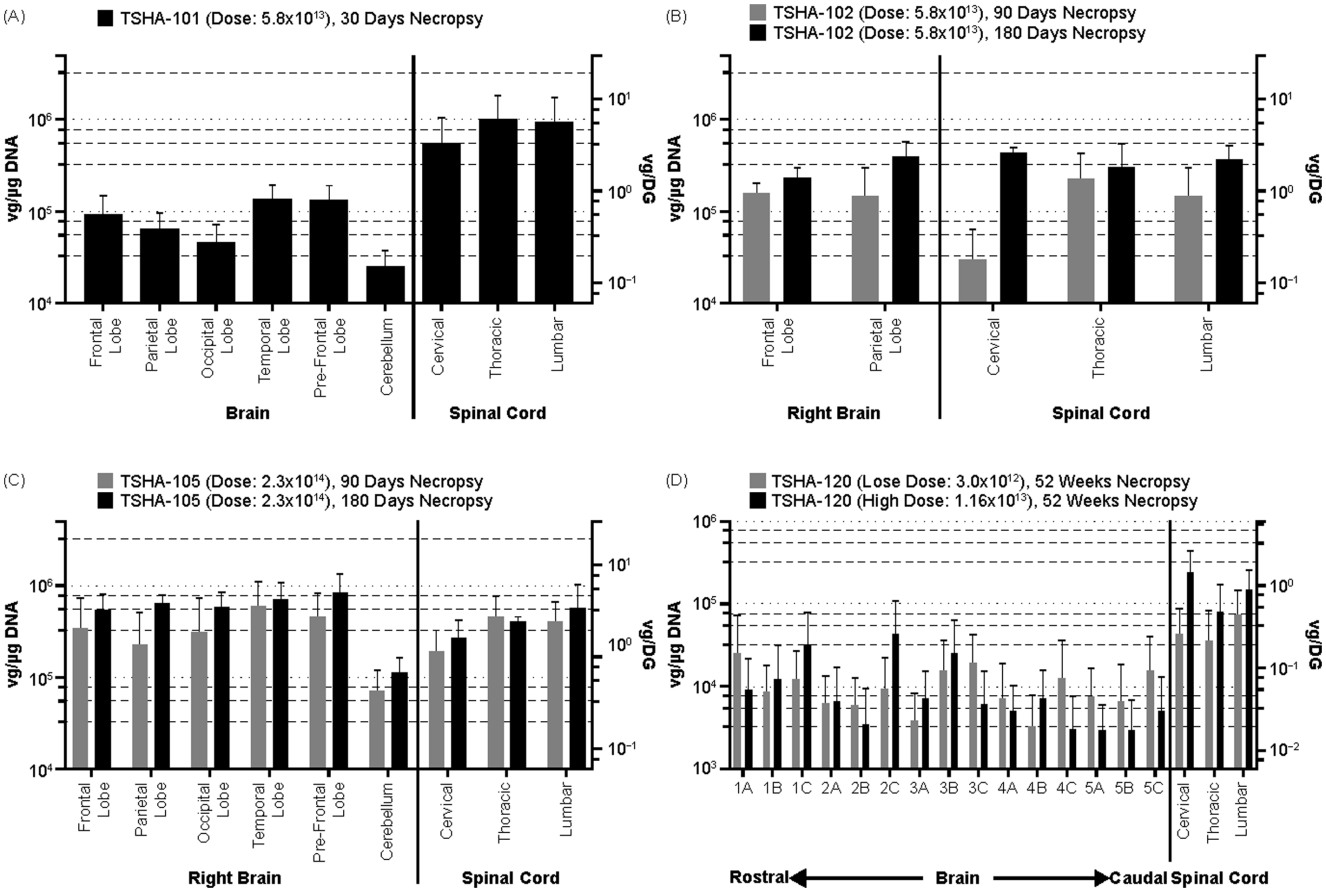
Brain and spinal cord biodistribution of rAAV9 vector genomes in NHPs following lumbar IT dosing. Graphs report mean ± SD vector genome values per μg genomic DNA in NHP CNS samples taken at the indicated necropsy time following administration at the indicated doses of: **(A)** TSHA-101 (*n* = 4); **(B)** TSHA-102 (*n* = 6, including three at the 90 day time point and three at 180 days); **(C)** TSHA-105 (*n* = 7, four at the 90 day time point and three at 180 days); and **(D)** TSHA-120 [*n* = 8 (four animals at each dose)]. CNS, central nervous system; DG, diploid genome; IT, intrathecal administration; NHP, nonhuman primate; SD, standard deviation; rAAV9, recombinant adeno-associated virus serotype 9; Rt., right; vg, vector genome.

Four NHPs treated with a single dose of TSHA-101 at 5.8 × 10^13^ vg/animal (HED: 5 × 10^14^ vg) were sacrificed at Day 30, and rAAV9 was quantified in six brain regions (prefrontal, frontal, parietal, occipital, and temporal lobes, as well as cerebellum) and three spinal cord regions (lumbar, thoracic, and cervical). Biodistribution was consistent across brain regions mean values ranging from ~2.5 × 10^4^ vg/μg (0.16 vg/DG) in the cerebellum to ~1.3 × 10^5^ vg/μg (0.8 vg/DG) in the prefrontal lobe. Biodistribution was also consistent across spinal cord samples, albeit at a higher level of ~8 × 10^5^ vg/μg (5 vg/DG) (Figure 1A).

### 3.2 TSHA-102 CNS biodistribution at 90 and 180 days post-lumbar IT administration

Six NHPs treated with a single dose of TSHA-102 at 5.8 × 10^13^ vg/animal (HED: 5 × 10^14^ vg) were sacrificed at Days 90 (*n* = 3) and 180 (*n* = 3). CNS biodistribution showed generally similar vector genome levels in all CNS tissues tested, with a low value observed at the 90-day point for the cervical spinal cord [~2.9 × 10^4^ vg/μg (0.118/DG)] (Figure 1B). Across brain regions, mean vector genome levels ranged from 1.5 × 10^5^ vg/μg (0.92 vg/DG) at Day 90 to 3.1 × 10^5^ vg/μg (1.87 vg/DG) at Day 180, reflecting a modest increase in vector genome levels observed at the later time point.

### 3.3 TSHA-105 at 90 and 180 days post-lumbar IT administration

Seven NHPs treated with a single dose of TSHA-105 at 2.3 × 10^14^ vg/animal (HED: 2.0 × 10^15^ vg) were sacrificed at Days 90 (*n* = 4) and 180 (*n* = 3). CNS biodistribution showed generally consistent vector genome levels among brain and spinal cord tissues (Figure 1C). As in the TSHA-102 data set, vector genome levels appeared greater at Day 180 than at Day 90. Across brain tissues, mean vector genome levels at Days 90 and 180 were 3.9 × 10^5^ vg/μg (2.4 vg/DG) and 6.7 × 10^5^ vg/μg (4.0 vg/DG), respectively. Comparing TSHA-102 versus TSHA-105 data, both with lumbar IT delivery, the four-fold increase in input dose with TSHA-105 was associated with a ~2.5-fold increase in vector genome levels detected in brain tissues; this difference was seen at both necropsy time points.

### 3.4 TSHA-120 CNS biodistribution at 1 year post-lumbar IT administration

In one biodistribution study, eight NHPs were treated with TSHA-120 at each of two doses (3 × 10^12^ and 1.2 × 10^13^ vg/animal) and were followed for 52 weeks. Notably, these vector genome levels, with an HED of 2.6 × 10^13^ vg and 1 × 10^14^ vg, respectively, are low compared to current expectations of rAAV9 GT directed to the CNS (e.g., up to 1 × 10^15^ vg per patient, with TSHA-102). Serial brain sections (five slabs along a rostro-caudal axis; three tissue samples per slab) showed quantifiable levels of vector genome in each sample, ranging from approximately 3.0 × 10^3^ to 4.2 × 10^3^ vg/μg (0.02 to 0.27 vg/DG) (Figure 1D). Vector biodistribution was generally consistent and did not vary appreciably across the rostro-caudal axis, from the brainstem to the prefrontal cortex; no consistent dose-dependence was evident over the 3.9-fold dose range applied. In spinal cord samples, values ranged from approximately 3.6 × 10^4^ to 2.4 × 10^5^ vg/μg (0.23 to 1.54 vg/DG).

### 3.5 TSHA-102 CNS biodistribution at 90 days post-ICM administration

**Figure 2:**
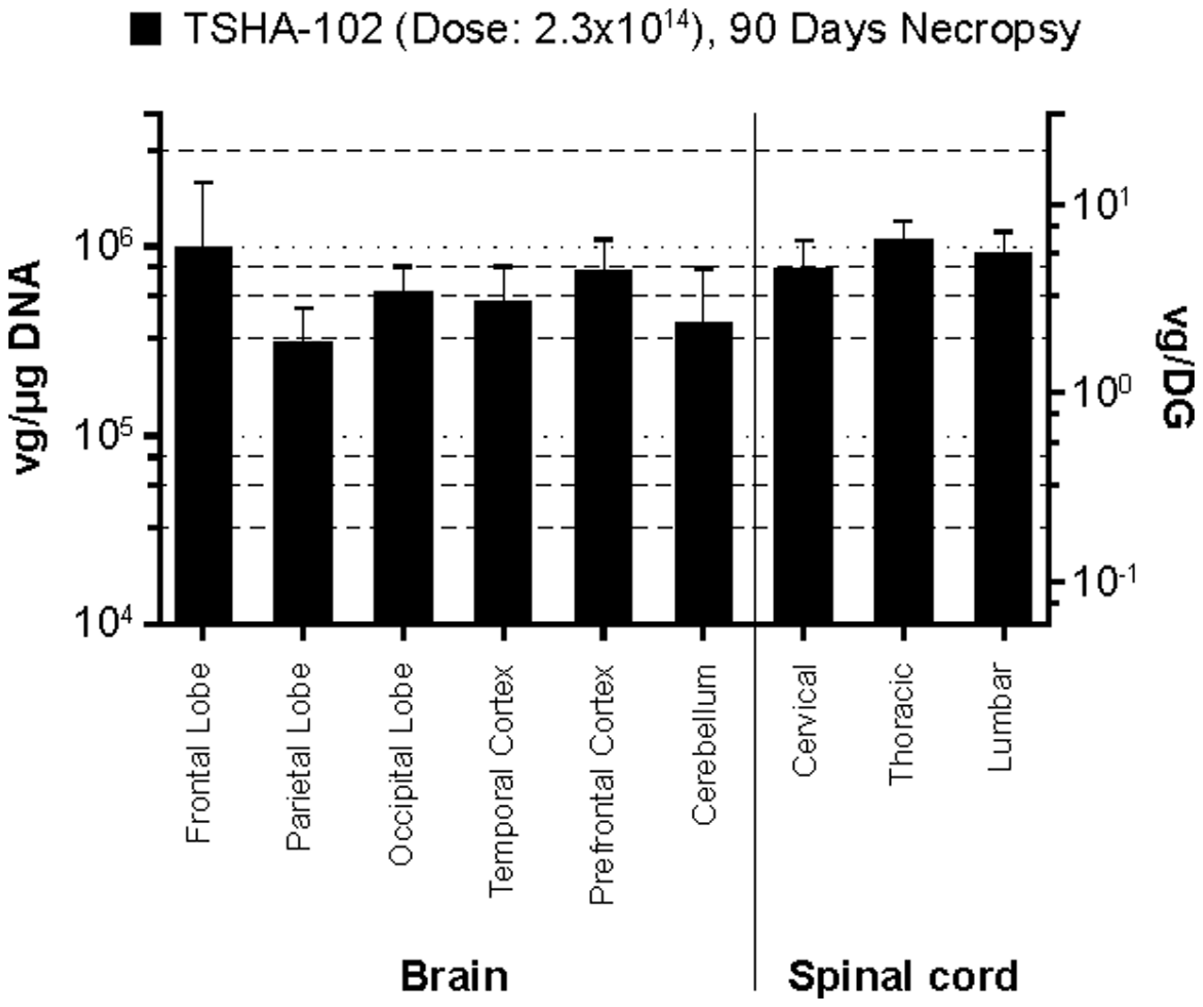
rAAV9 vector genome biodistribution at necropsy Day 90 following ICM dosing (2.3 × 10^14^ vg/animal). DG, diploid genome; Rt., right; vg, vector genome.

ICM dosing was investigated in three NHPs treated with a single dose of TSHA-102 at 2.3 × 10^14^ vg/animal (HED: 2 × 10^15^ vg) and sacrificed at Day 90. rAAV9 was quantified in six brain regions (prefrontal, frontal, parietal, occipital, and temporal lobes, as well as cerebellum) and three spinal cord regions (lumbar, thoracic, and cervical). Biodistribution was consistent across brain and spinal cord regions [mean values ranging from ~5.8 × 10^5^ vg/μg (3.5 vg/DG) in the brain to ~9.2 × 10^5^ vg/μg (5.5 vg/DG) in the spinal cord] (Figure 2).

**Figure 3:**
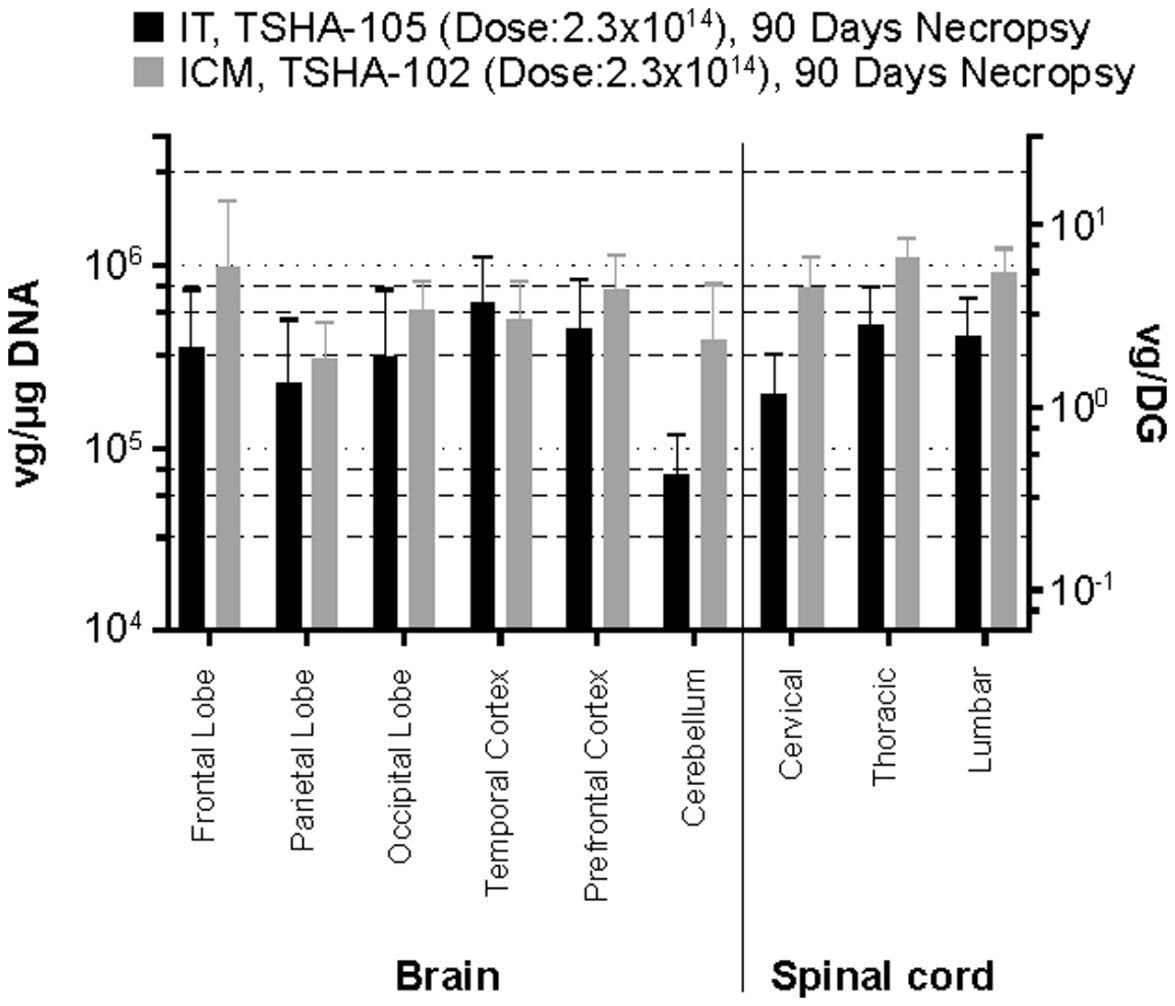
Comparison of rAAV9 biodistribution following equivalent dosing (2.3 × 10^14^ vg/animal) by lumbar IT (TSHA-105) and ICM (TSHA-102) administration at 90 days post-dosing. DG, diploid genome; ICM, intracisterna magna administration; IT, intrathecal administration; Rt., right; vg, vector genome.

Figure 3 shows comparative biodistribution of TSHA-102 following ICM administration versus TSHA-105 following lumbar IT administration. The rAAV9 doses applied were identical, and all tissues were examined following a Day 90 necropsy. Across the six brain regions and three spinal cord regions examined, lumbar IT and ICM delivery led to similar mean vector genome levels, typically ~5.0 × 10^5^ vg/μg (~3 vg/DG). The ratio of ICM to lumbar IT vector biodistribution in various brain and spinal cord tissues ranged from <1 (temporal lobe) to ~5 (cerebellum).

### 3.6 Dose-dependence and temporal effects on rAAV9 levels in the brain

**Figure 4:**
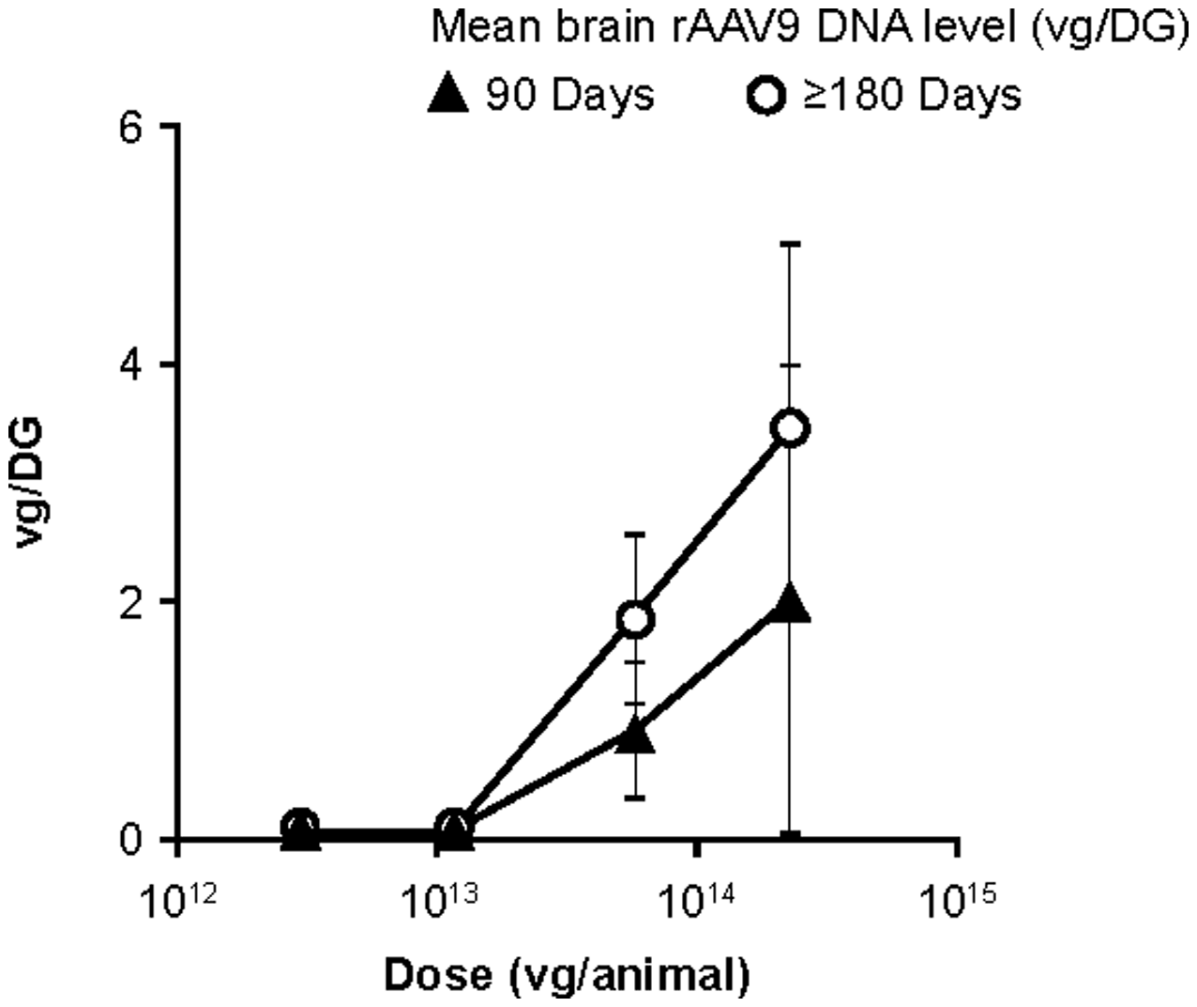
Dose-dependent rAAV9 levels in the brain across lumbar IT dosing studies. Each data point corresponds to a minimum of six brain tissue samples. For instance, for the right-most triangle on the graph, mean and vg/DG were calculated from the vg/DG values for 24 tissue samples (six brain samples for each of four animals receiving TSHA-105 lumbar IT at 2.3 × 10^14^ vg, necropsied at Day 90). DG, diploid genome; IT, intrathecal administration; rAAV9, recombinant adeno-associated virus serotype 9; vg, vector genome.

Since comparisons within and across the four lumbar IT studies suggested that rAAV9 delivery was generally dose-dependent for the various brain regions when considered independently, a *post-hoc* analysis was conducted, incorporating rAAV9 DNA levels from all brain regions from animals with scheduled necropsies at Day 90 or Day ≥180 after lumbar IT administration. Mean level of rAAV9 DNA in the brain corresponded to 1 to 4 vg/DG at all doses ≥5.8 × 10^13^ vg/animal (HED: 5.0 × 10^14^ vg), with an apparent dose-dependence seen both in the Day 90 brain samples and the Day ≥180 brain samples (Figure 4).

**Figure 5:**
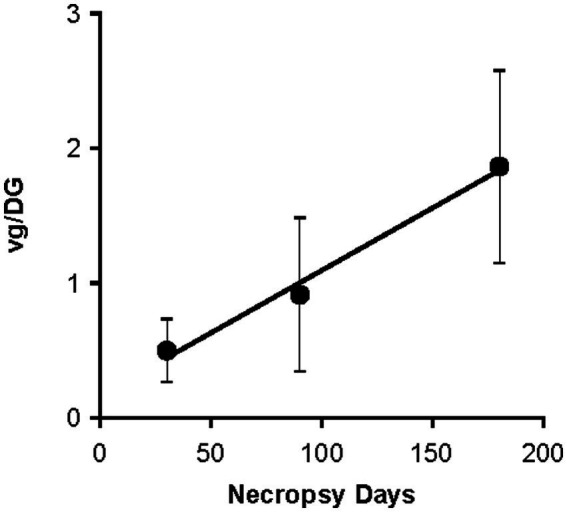
Time-dependent rAAV9 biodistribution in the brain following rAAV9 dosing at 5.8 × 10^13^ vg/animal, lumbar IT dosing. The Day 90 and 180 data points each correspond to the mean (SD) vg/DG value for six brain slices. The Day 30 time point was calculated from 24 brain samples (six from each of four animals treated with TSHA-101). DG, diploid genome; IT, intrathecal administration; rAAV9, recombinant adeno-associated virus serotype 9; SD, standard deviation; vg, vector genome.

This analysis also indicated higher brain vector genome levels in Day ≥180 necropsy samples, relative to Day 90 samples, in animals that had been treated by lumbar IT dosing. This apparent time-dependence was examined separately, again using pooled rAAV9 levels from tissue samples throughout the brain. At a single lumbar IT dose level (5.8 × 10^13^ vg/animal; HED: 5.0 × 10^14^ vg), mean brain rAAV9 DNA appeared to increase linearly between Day 30 and Day 180 post-dosing (Figure 5).

## 4 Discussion

The four GT constructs described here share a common AAV9 serotype but differ with respect to their therapeutic gene of interest and viral genomic structure (self-complementary for three of the vectors; single-stranded in the case of TSHA-101). In contrast to serotype, which clearly affects cell tropism, genomic structure is unlikely to influence the initial kinetics of vector uptake by target cells (10). After an AAV virion is internalized and transported to the nucleus and viral DNA is released, cell transduction would ordinarily depend on host cell factors to carry out second-strand biosynthesis (11). Use of scAAV9 vectors avoids the need for this rate-limiting step and is associated with greatly increased transduction efficiency— at least a 10-fold improvement in brain cell transduction and a corresponding increase in transgene expression, relative to ssAAV9 vectors (12, 13).

Lumbar IT injection, a minimally invasive RoA facilitating direct CNS access, was used in most cases. As with ICM and ICV injection, lumbar IT injection reduces systemic exposure and sidesteps concerns about neutralizing antibodies, while allowing for considerable flexibility in dosing (2, 3). Uniquely, it offers the possibility of outpatient treatment with no specialized surgical equipment. However, some studies have suggested the lumbar IT route leads to limited transduction of brain tissues (5, 14).

Across four NHP biodistribution studies, at times ranging from 1 month to 1 year post-treatment, lumbar IT administration led to widespread, stable, and generally uniform vector genome levels throughout the brain and spinal cord. Within and across studies, vector genome levels in brain and spinal cord tissues were generally proportional to the rAAV9 vector dose, although at the lowest dose range studied (3 × 10^12^ to 1.2 × 10^13^ vg/animal for TSHA-120; a 3.9-fold input dose range), no consistent trend was observed in TSHA-120 DNA levels in the brain or spinal cord. In contrast with IT and ICM treatment at higher rAAV9 doses, the lack of dose-dependence in this study has not been explained but may reflect limited assay sensitivity.

rAAV9 biodistribution showed comparable results following equivalent dosing (2.3 × 10^14^ vg/animal; HED: 2.0 × 10^15^ vg) by lumbar IT (TSHA-105) versus ICM (TSHA-102) administration at 90 days post-dosing. Figure 3 shows that both the lumbar IT and ICM RoA led to widespread and consistent biodistribution across brain and spinal cord tissues, at comparable mean levels of ~4 × 10^5^–6 × 10^5^ vg/μg (2.4–3.5 vg/DG) and 3.6 × 10^5^–9.2 × 10^5^ vg/μg (2.2–5.5 vg/DG). Analysis of TSHA-102 and TSHA-105 biodistribution, following dosing at 5.8 × 10^13^ vg/animal (HED: 5.0 × 10^14^ vg) and 2.3 × 10^14^ vg/animal (HED: 2.0 × 10^15^ vg), respectively, suggests that a four-fold higher dose of TSHA-105 led to 1.5–2.5- fold higher rAAV9 levels across different tissues. Similarly, comparing the two studies of TSHA-102, ICM dosing at 2.3 × 10^14^ vg/animal (4-fold higher than the lumbar IT-administered dose of TSHA-102), led to consistently greater vector genome levels across CNS tissues by a factor of ~4-fold. These findings are consistent with a dose-dependent effect at this range and suggest no substantial difference in rAAV9 DNA levels in the brain, comparing lumbar IT versus ICM delivery routes.

Interestingly, the rAAV9 DNA levels reported here are in proportion to those originally reported by Gray et al. (4) for transduction in the NHP brain. Using a 100-fold lower rAAV9 dose, those authors observed rAAV9 brain levels approximately 100-fold lower than those reported here. For comparison, the current clinical protocol for therapeutic use of TSHA-102 in RTT calls for lumbar IT dosing at 1 × 10^15^ vg. These findings, together with the data presented here, show a proportional biodistribution profile relative to the administered rAAV9 dose.

In addition to the apparent dose effect, we noted an unexpected but consistent increase in vector genome levels in brain tissues, related to time to necropsy. The dual effect of dosage and time since lumbar IT dosing can be seen by comparing Figures 1B,C, examining TSHA-102 and TSHA-105 biodistribution from two studies with 90- and 180-day necropsy times. This comparison is shown in Figures 4, 5, in which brain tissue data are pooled, rather than being examined separately for different brain regions.

No such temporal effect has been reported previously, and it is possible it represents a chance finding; Hordeaux et al. (15) reported no such effect after 90 days versus 180 days in rhesus macaques dosed by the ICM route. Regardless, our data confirm that rAAV9 DNA is present throughout the brain as well as the spinal cord over the time courses studied, up to at least 1 year after treatment (Figure 1D). The sustained presence of rAAV9 DNA may support the long-term therapeutic benefit from a single administration.

A key observation in our studies relates to the apparently uniform distribution of rAAV9 throughout regions of the brain. Both the lumbar IT and ICM RoAs appeared to deliver rAAV9 virions effectively throughout the brain parenchyma, with little difference in mean biodistribution between brain regions, including the brain stem, the frontal, pre-frontal, temporal, occipital, and parietal lobes, and the cerebellum. Of these, biodistribution to the cerebellum was moderately reduced, relative to other brain tissues, for TSHA-101 (IT), TSHA-102 (IT and ICM) and TSHA-105 (IT). Other groups have previously reported relatively reduced biodistribution of rAAV to the cerebellum, compared with other CNS tissues, following IT (5) or ICM (15) administration. Despite the apparent difficulty of transducing the cerebellum, lumbar IT dosing with TSHA-105 at a HED of 2 × 10^15^ vg was associated with mean cerebellar biodistribution of ~0.7 vg/DG; with the rAAV9 dose used in this experiment, all other brain tissues maintained still-higher vector genome levels, e.g., ~1–2 vg/DG.

The ICM route was selected as a mechanistic comparator to the IT route, allowing cerebrospinal fluid (CSF) entry site to be evaluated for possible influence on vector biodistribution. However, the differences observed in vector biodistribution with lumbar IT versus ICM dosing can be explained by a simple dosage effect, such that a 4-fold higher dose delivered by the ICM route led to a ≤4-fold higher vector genome level in the brain at Day 90, relative to lumbar IT dosing.

Our observation of consistent rAAV9 biodistribution throughout the brain with both of these RoAs challenges some reports suggesting that virions have limited access to brain tissues following lumbar IT dosing, relative to other CSF-directed RoAs. Hinderer et al. (14) have proposed that ICM dosing could be up to two orders of magnitude more effective for GT of the brain and spinal cord, relative to lumbar IT dosing. Such a difference between RoAs has not been explained, although a recent review on intra-CSF AAV studies (6) suggests several possible reasons, including simple technical variability in the injection methods. For example, Ohno et al. (16) visualized the fluid kinetics of contrast-mixed AAV9, injected intrathecally in NHPs, and observed rapid leakage of most of the vector solution out of the IT space from the injection site in two-thirds of the animals.

As Hinderer et al. (14) note, their finding appears at odds with observations of exogenous proteins delivered to the brain through the CSF. Specifically, pharmacokinetic studies of lumbar IT dosing suggest that this RoA efficiently delivers therapeutic proteins throughout the brain parenchyma and confirm that this process is dose-responsive and rapid, with protein detectable in white and grey matter within 1 h and maximal by 6 h (17, 18). While no direct comparison of lumbar IT and ICM protein administration appears to have been reported, Calais et al. (19) have observed comparable delivery and brain distribution of a therapeutic enzyme in cynomolgus macaques following either lumbar IT or ICV administration, suggesting that bulk glymphatic flow after lumbar IT dosing rapidly distributes this protein cargo throughout the NHP brain.

It is possible that cargoes differ in this regard (5, 16, 19), or that differences in animal age or health, or subtle variations in lumbar IT instillation methodology (20) could contribute to inconsistent findings regarding rAAV9 biodistribution. Although higher-resolution approaches would be required to provide more detail and cell-type specificity, our experience with various AAV9-based vectors supports the assumption that all regions of the brain are treated, with coverage on the order of ~1 vg per brain cell following dosing at 5.8 × 10^13^ vg/animal (HED: 5.0 × 10^14^ vg). Moreover, the lumbar route is clearly less invasive than direct intraparenchymal dosing. As others (2, 19) have observed, lumbar IT dosing is less demanding of specialized surgical approaches, relative to RoAs that target the CSF at sites adjacent to the brain, which therefore carry some risk of trauma to critical structures in the brainstem or medulla.

### 4.1 Strengths and limitations

To our knowledge, this is the first retrospective analysis of vector biodistribution to aggregate vector genome data from NHPs treated with the same serotype (rAAV9) but different therapeutic transgenes. Because the AAV9 virion structures were identical and dosing procedures were similar across the four lumbar IT studies, it was expected that NHP brain biodistribution would be comparable, independent of the payload, as was indeed observed here. The 28 rAAV9-treated animals examined here for brain biodistribution provide a robust data set for quantitative analysis of vector delivery in a large-animal GT model. The fact that these studies were conducted separately, according to different protocols, limits the precision of any quantitative claims that can be made for rAAV9 biodistribution from this data set, but in our view it does not weaken the key qualitative conclusion that all four rAAV9 vector genome were detected at generally uniform levels throughout the brain. Conversely, the use of two distinct RoAs for a single vector, TSHA-102, represents an important strength of the current report.

In addition, the available studies allow comparison only between the lumbar IT and ICM routes; the vectors used here have not been administered via other intra-CSF RoAs, such as ICV dosing in a large-animal model. In a canine model of mucopolysaccharidosis I, Hinderer et al. (5) found that the efficiency of brain delivery was similar for dogs treated with a rAAV9 GT vector (2.9 × 10^13^ vg/animal), by the ICM versus ICV routes (*n* = 3 animals for each RoA). Similar to our observations with lumbar IT and ICM dosing in NHPs, Hinderer et al. (5) found that the biodistribution of vector genomes was ≤1 vg/DG throughout the brain, irrespective of RoA, although rAAV9 levels were somewhat higher (on the order of 10 vg/DG) with ICM dosing in the cervical and thoracic spinal cord. However, the authors noted a stark difference between the ICM and ICV approaches with regard to safety; ICV-dosed dogs experienced a strong (and, in one case, fatal) encephalitis, apparently driven by T cells specific to the transgene product, but no such response was seen in ICM-treated animals. The relevance of the canine safety and biodistribution data to NHP and human GT is not certain.

Analysis was restricted to vector biodistribution, rather than gene or protein expression, because the vectors all differed in their promoter and other regulatory sequences, precluding any comparison across experiments. For the two NHP studies involving TSHA-102, gene expression is expected to be post-transcriptionally repressed in wild-type (i.e., MeCP2-expressing) brain tissue, as has indeed been observed in NHPs and other animal and cell models (7, 21, 22). TSHA-102 carries a microRNA-responsive regulatory element (miRARE), which was designed to silence expression of the of the mini*MECP2* transgene cell autonomously in MeCP2^+^ cells, due to miRNA-dependent mRNA decay and/or translational silencing. Hence, RNA expression was not evaluated in this report.

Finally, whereas the generally uniform and dose-dependent biodistribution we report speaks to the ability of IT-delivered rAAV9 to access different brain tissues, mean vg/DG must not be understood as an estimate of the proportion of transduced cells. Among other concerns, neuronal and non-neuronal cell-type differences may affect the efficiency of rAAV9 uptake, and rAAV9 DNA detection offers no assurance that the vector genome is fully intact or that it has been internalized into the cell and cell nucleus.

## 5 Conclusion

Across five NHP biodistribution studies, rAAV9 DNA was quantifiable in all brain and CNS tissues examined, irrespective of RoA (IT versus ICM) or time post-necropsy, at typical levels exceeding 1 vg copy per brain cell at the highest doses. These findings support the use of IT dosing as a safe, effective, and minimally invasive approach to GT for the treatment of genetic CNS diseases. rAAV9 doses explored in these preclinical experiments are comparable to those currently under clinical investigation in women and girls with Rett syndrome in the REVEAL adult/adolescent (NCT05606614) and pediatric (NCT06152237) phase 1/2 trials.

Early observations from the REVEAL Phase 1/2 first-in-human clinical trials evaluating the safety and preliminary efficacy of a single IT administration of TSHA-102 indicate that reported participants dosed in these studies have experienced improvements across the core domains impacted by RTT, including gain or regain of one or more developmental milestones, with improvement noted in autonomic function, communication and social interaction, and gross and fine motor function (23). These findings align with the available preclinical data and published literature, indicating that IT dosing results in widespread distribution of TSHA-102 and other rAAV9 DNA throughout the brain, supporting functional improvements in multiple regions such as the motor cortex, cerebellum, brainstem, and prefrontal cortex.

## Data Availability

The original contributions presented in the study are included in the article/Supplementary material.

## Supplementary material

SUPPLEMENTARY FIGURE S1 Two common CNS-directed routes of administration for rAAV9 gene therapy. In both ICM and lumbar IT administration, virions are administered in the CSF and delivered to the brain via glymphatic flow. In the work described here, four different rAAV9 constructs were dosed by the lumbar IT route, and one of the constructs (TSHA-102) was also delivered by the ICM route. CNS, central nervous system; CSF, cerebrospinal fluid; ICM, intracisterna magna administration; IT, intrathecal administration; rAAV9, recombinant adeno-associated virus serotype 9.
