# Supplementary figures and images for: rAAV9 vector biodistribution in nonhuman primate brain and spinal cord following lumbar intrathecal infusion
Source: Front Med (Lausanne). 2026 Apr 29;13:1819594. doi: 10.3389/fmed.2026.1819594 (PMC13167494; doi:10.3389/fmed.2026.1819594)

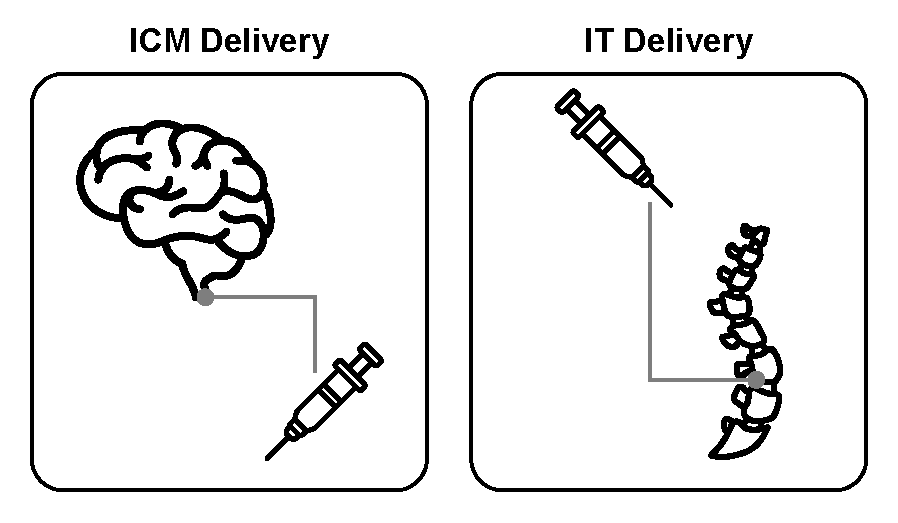

Supplement: SUPPLEMENTARY FIGURE S1 — Two common CNS-directed routes of administration for rAAV9 gene therapy. In both ICM and lumbar IT administration, virions are administered in the CSF and delivered to the brain via glymphatic flow. In the work described here, four different rAAV9 constructs were dosed by the lumbar IT route, and one of the constructs (TSHA-102) was also delivered by the ICM route. CNS, central nervous system; CSF, cerebrospinal fluid; ICM, intracisterna magna administration; IT, intrathecal administration; rAAV9, recombinant adeno-associated virus serotype 9. [file Image_1.TIF]
